# Clinical outcomes of single-step transepithelial photorefractive keratectomy and off-flap epipolis-laser in situ keratomileusis in moderate to high myopia: 12-month follow-up

**DOI:** 10.1186/s12886-022-02443-6

**Published:** 2022-05-23

**Authors:** Yunjie Zhang, Tiankun Li, Zhangliang Li, Mali Dai, Qinmei Wang, Chenchen Xu

**Affiliations:** 1 Eye Hospital and School of Ophthalmology and Optometry, Wenzhou Medical University, Wenzhou, Zhejiang China; 2 National Clinical Research Center for Ocular Diseases, Wenzhou, Zhejiang China; 3 Eye Hospital of Wenzhou Medical University Hangzhou Branch, 270 West Xueyuan Road, Wenzhou, 325027 Zhejiang China

**Keywords:** Transepithelial photorefractive keratectomy, Epipolis-laser in situ keratomileusis, Higher-order aberrations

## Abstract

**Background:**

To compare the quantitative and qualitative optical outcomes of single-step transepithelial photorefractive keratectomy (TPRK) and off-flap epipolis-laser in situ keratomileusis (Epi-LASIK) in moderate to high myopia.

**Methods:**

In this prospective self-control study, we included patients with moderate to high myopia who were randomized to undergo TPRK in one eye and Epi-LASIK in the other eye. Twelve-month follow-up results for visual acuity, refraction, ocular high-order aberrations, contrast sensitivity, postoperative pain, epithelial healing, and haze grade were assessed.

**Results:**

A total of 64 eyes (32 patients) were enrolled in the study. More eyes completed re-epithelialization in the TPRK group than in the Off-flap Epi-LASIK group 3–4 days postoperatively, while all eyes completed re-epithelialization by 7 days. More eyes achieved a visual acuity (both UDVA and CDVA) of better than 20/20 in the TPRK group than in the Off-flap Epi-LASIK group. The ±0.50 D predictability for correction of the spherical equivalent (SE) was higher in the eyes of the TPRK group (91%) than in those of the off-flap Epi-LASIK group (80%) 12 months after surgery. No significant differences in ocular aberrations, including coma, spherical, and trefoil, were found between the two groups at 12 months. There were also no significant differences in visual acuity, contrast sensitivity, pain, and haze grading between the two groups.

**Conclusions:**

Both TPRK and off-flap Epi-LASIK are safe, effective, and predictable treatments for moderate to high myopia with comparable surgical outcomes.

**Trial registration:**

This study was retrospectively registered on ClinicalTrial.gov (NCT05060094, 17/09/2021).

**Supplementary Information:**

The online version contains supplementary material available at 10.1186/s12886-022-02443-6.

## Background

Photorefractive keratectomy (PRK) and laser in situ keratomileusis (LASIK) were introduced decades ago [1, 2], and with recent advances in surgical techniques and adjunctive mitomycin-C medication, the outcome of modern surface ablation is excellent and comparable to that of LASIK [3, 4].

A key aspect of modern surface ablation is epithelial removal. Laser-assisted sub-epithelial keratectomy (LASEK), a technique which allows for the separation of the epithelium using 20% alcohol, was introduced in 2001 [5]. And, in the same year, epipolis-laser in situ keratomileusis (Epi-LASIK) was developed as an alternative to epithelial separation using a modified microkeratome [6]. Recently, Feng et al. [7] demonstrated that complete removal of the epithelial flap in Epi-LASIK (off-flap) resulted in less postoperative pain, more rapid re-epithelialization, and faster visual recovery compared to flap-reserved Epi-LASIK (on-flap).

Another alternative technique for epithelial removal is transepithelial PRK (TPRK), in which epithelial removal is performed with laser phototherapeutic (PTK) ablation, followed by laser refractive ablation of the stroma. However, several studies have reported that two-step TPRK using different laser platforms produces variable results [8–11]. Excitingly, a single-step TPRK was recently introduced which allows for simultaneous ablation of both the epithelium and stroma which shortens treatment time, minimizes the risk of corneal dehydration, and prevents any instrument to eye contact. This new technique uses a population-based epithelial thickness ablation profile of 55 μm centrally, and 65 μm at 4 mm radially from the center to match the area of the total ablation zone. Considering that the water content of the epithelium is different from that of the stroma, the ablation rates of these two are also different. While previous studies have compared the outcomes between single-step TPRK and alcohol-assisted PRK, no study to date has assessed the outcomes between single-step TPRK and off-flap epi-LASIK [12–14].

Therefore, in this study, we evaluated the long-term outcomes of single-step TPRK compared to off-flap epi-LASIK in moderate to high myopia, including qualitative and quantitative parameters, and performed vector analysis of astigmatism.

## Methods

### Patients and methods

Patients with myopia (up to − 9.75 D) with or without astigmatism (up to 3.5 D) were enrolled in a prospective interventional study. The study was approved by the Wenzhou Medical University Institutional Review Board and adhered to the tenets of the Helsinki Declaration. Patients received a full explanation of both procedures and provided written informed consent. Patients were randomized to have TPRK in one eye and Epi-LASIK in the other eye using a random number table generated by Microsoft Excel.

Inclusion criteria included: 18 years of age or older; a corrected distance visual acuity (CDVA) in logarithm of minimal angle of resolution (LogMAR) of 0.10 or better; refractive error stabilized for at least 1 year; and discontinuation of contact lens use for at least 2 weeks. Exclusion criteria included: the presence of corneal scars, keratoconus, glaucoma, retinal diseases, or a history of corneal or intraocular surgery.

### Patient assessment

All patients underwent comprehensive ophthalmological examinations, including uncorrected (UDVA) and corrected distance visual acuities (CDVA), manifest and cycloplegic refraction preoperatively, intraocular pressure, an anterior and posterior segment examination, corneal topography measured by a Scheimpflug scanning-slit topographer (Pentacam, Oculus Optikgerate GmbH, Wetzlar, Germany), and contrast sensitivity under photopic and mesopic conditions (CSV-1000E, Vector Vision Inc., Greenville, OH, USA) with correction by spectacles and ocular wavefront aberrometry (OPD-Scan II, Nidek Co. Ltd., Japan). These examinations were repeated 1, 3, 6, and 12 months after surgery.

### Surgical technique

Both single-step TPRK and off-flap Epi-LASIK were performed with the Amaris 750 S (Schwind eye-tech-solutions, Germany) using integrated ORK-CAM software, with the right eye operated on first in all cases. Local anesthetic was applied for the ablation procedure, using proparacaine hydrochloride 0.5% drops (Alcaine, Alcon, USA) three times every 5 min.

In the single-step TPRK procedure, transepithelial corneal epithelial removal was performed using the ORK-CAM software module mode followed by stromal excimer laser ablation in a single step based on an aberration-free and aspheric profile. The area of epithelial removal was defined as the area of the total ablation zone. The software compensated for the energy loss at the slope of the peripheral cornea. In the off-flap Epi-LASIK group, the epithelium was removed using an epikeratome (Moria, Antony, France) with a diameter of 9 mm, the stromal ablation was performed with Amaris 750 s excimer laser. After ablation, 0.02% mitomycin-C was applied to the stromal bed for 15–30 s and then rinsed with a balanced salt solution. A bandage contact lens (Acuvue Oasis, Johnson & Johnson, USA) was placed on the cornea at the end of the surgery. All procedures were performed by the same surgeon (XCC).

### Postoperative medication and assessment

Patients were prescribed levofloxacin 0.5% eyedrops (Cravit; Santen, Japan) and pranoprofen 0.1% eye drops (Senju Pharmaceutical Co., Ltd., Japan) four times a day for the first 3 days after surgery. After removing the contact lens, tobramycin-dexamethasone eye drops (Tobradex; Alcon, Ltd. USA) were administered four times a day for 2 weeks, followed by 0.1% fluorometholone eye drops (FML; Allergan, Irvine, CA) which were used four times a day for the next 2 weeks, after which they were tapered every week to once daily. Preservative-free artificial tears every 2 hours were prescribed until complete re-epithelialization, and then reduced to four times a day for 6 months.

Epithelial healing was observed daily from the third postoperative day, and the contact lens was removed when corneal re-epithelialization was completed. Subjective pain scores were evaluated on the third day according to a predetermined scale ranging from 0 to 5 as follows: 0, no pain or discomfort; 1, photophobia and tears; 2, photophobia and tears with mild pain; 3, photophobia and tears with moderate pain that does not require oral medication; 4, photophobia and tears with severe pain that oral medication can relieve; 5, photophobia and tears with severe pain that oral medication cannot relieve. The corneal haze grade was recorded according to the methods reported by Fantes et al. [15]: 0, no haze; 0.5, trace haze that could only be seen by oblique illumination; 1, a more visible haze not interfering with the visibility of iris details; 2, mild influence of iris details; 3, moderate influence of iris details; 4, marked haze obscuring the stroma of the ablation area.

### Statistical analysis

Statistical analysis was performed using SPSS software (version 22.0, SPSS, Chicago, Inc.). The Kolmogorov-Smirnov test was used to check the normal distribution of the variables. Student’s t-test or Wilcoxon rank sum test was used based on the normality of the data. Statistical significance was set at *p* < 0.05.

## Results

In total, 38 patients were initially enrolled in the study. Thirty-two patients finally completed the 12-month follow-up, and six patients were lost to follow-up. No significant difference was noted between the groups at baseline with respect to spherical equivalent (SE), CDVA, central corneal thickness (CCT), or epithelial thickness. The optical zones between the groups were not significantly different. However, the central ablation depth and total ablation zone were significantly higher in the TPRK group compared to the Off-flap Epi-LASIK group (both *P* < .001, Table 1).

**Table 1:** Baseline characteristics of eyes that had transepithelial PRK or off-flap Epi-LASIK

	Transepithelial PRK		Off-flap Epi-LASIK		*P* value
Parameter	Mean ± SD	Range	Mean ± SD	Range	
Refractive errors (D)					
Sphere	−6.03 ± 1.97	−9.50, −2.50	− 6.18 ± 2.01	−9.75, − 2.50	.363
Cylindrical	−1.09 ± 0.72	−3.00, 0.00	− 1.03 ± 0.73	− 3.50, 0.00	.616
SE	−6.57 ± 2.01	−10.00, − 2.88	− 6.67 ± 1.99	− 10.25, − 2.88	.519
UDVA (logMAR)	1.07 ± 0.22	0.50, 1.60	1.08 ± 0.23	0.50, 1.60	.730
CDVA (LogMAR)	−0.003 ± 0.05	−0.10, 0.10	0.003 ± 0.04	−0.10, 0.10	.423
CCT (um)	498.78 ± 23.81	465.00, 570.00	499.00 ± 22.53	462.00, 556.00	.789
Total ablation zone (mm)	7.95 ± 0.32	6.70, 8.44	7.47 ± 0.26	6.89, 8.14	<.001
Optical zone (mm)	6.30 ± 0.32	5.80, 7.00	6.30 ± 0.35	5.80, 7.00	.872
Stromal ablation depth (um)	99.58 ± 23.18	44.00, 133.00	101.19 ± 19.40	61.00, 129.00	.232

*SE* Spherical equivalent, *UDVA* Uncorrected distance visual acuity, *CDVA* Corrected distance visual acuity, *logMAR* Logarithm of the minimum angle of resolution, *CCT* Central corneal thickness

### Corneal epithelial healing and pain score

More eyes completed re-epithelialization in the TPRK group 3 days (20 eyes, 63% vs. 16 eyes, 50%) and 4 days (25 eyes, 81% vs. 24 eyes, 78%) after surgery than in the off-flap Epi-LASIK group. Nevertheless, all eyes underwent re-epithelialization by 7 days. There was no significant difference in median pain score (range) between the two groups (TPRK: 2.0 [4] vs. Off-flap Epi-LASIK: 1.0 [5], *P* = .372).

### Visual acuity, efficacy, and safety

**Fig. 1:**
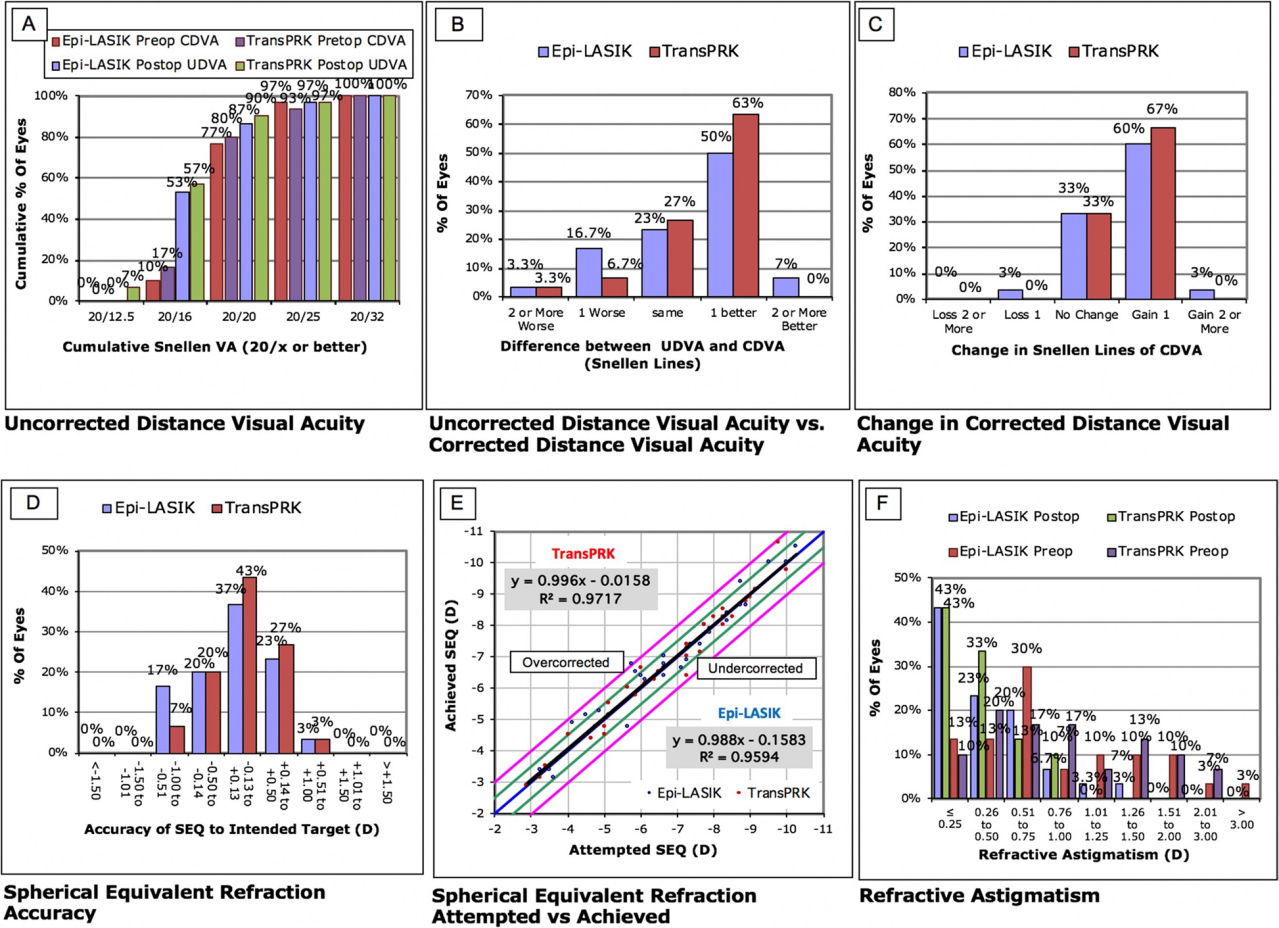
Visual outcomes after single-step transepithelial photorefractive keratectomy (TPRK) and off-flap epipolis-laser in situ keratomileusis (Epi-LASIK). **A**: cumulative 12-month postoperative uncorrected distance visual acuity (UDVA) and corrected distance visual acuity (CDVA); Changes in the Snellen lines of postoperative UDVA (**B**) and CDVA (**C**), compared with preoperative CDVA; **D**: accuracy of spherical equivalent refraction; **E**: attempted versus achieved changes in spherical equivalent refraction; **F**: distribution of preoperative and 12-month postoperative cylinder

During the period of postoperative observation, more eyes achieved visual acuity (both UDVA and CDVA) better than 20/20 in the TPRK group than in the Off-flap Epi-LASIK group (shown in Fig. 1A). None of the eyes lost two lines or more of CDVA in either group at the 12-month follow-up, and 60.00% (*n* = 18) of the eyes in the TPRK group and 63.33% (*n* = 19) eyes in the off-flap Epi-LASIK group gained one or two lines of improved CDVA (shown in Fig. 1B). There was no significant difference between the two groups in terms of line gain or loss (*P* = .58). Furthermore, no significant differences in UDVA or CDVA were noted between the groups (shown in Fig. 1C). At 12 months postoperatively, there were no significant differences in the mean efficacy index (ratio of postoperative UDVA to preoperative CDVA), mean safety index (ratio of postoperative to preoperative CDVA), refractive error (sphere, cylinder, and SE), UDVA, and CDVA between the two groups (Table 2).

**Table 2:** Comparison of postoperative visual acuity and refractive errors in eyes that had transepithelial PRK or off-flap Epi-LASIK

	Transepithelial PRK		Off-flap Epi-LASIK		*P* Value
Parameter	Mean ± SD	Range	Mean ± SD	Range	
Refractive errors (D)					
Sphere	0.21 ± 0.33	−0.50, 1.00	0.33 ± 0.46	−0.50, 1.25	.218
Cylindrical	−0.43 ± 0.30	−1.00, 0.00	−0.48 ± 0.39	−1.50, 0.00	.584
SE	−0.01 ± 0.35	−0.88, 0.88	0.08 ± 0.42	−0.88, 1.00	.311
UDVA (logMAR)	−0.05 ± 0.09	−0.20, 0.20	− 0.04 ± 0.08	−0.10, 0.20	.326
CDVA (logMAR)	−0.06 ± 0.06	−0.10, 0.10	− 0.05 ± 0.06	−0.10, 0.10	.522
Efficacy index	1.13 ± 0.16	0.79, 1.26	1.11 ± 0.19	0.79, 1.41	.733
Safety index	1.15 ± 0.12	1.00, 1.26	1.14 ± 0.13	0.89, 1.41	.631

*SE* Spherical equivalent, *UDVA* Uncorrected visual acuity, *CDVA* Corrected distance visual acuity, *logMAR* Logarithm of the minimum angle of resolution

### Refraction

The ±0.50 D predictability for correction of the spherical equivalent (SE) was higher in the eyes of the TPRK group (91%) compared to those of the off-flap Epi-LASIK group (80%) 12 months after surgery (shown in Fig. 1D). The linear regression model of the attempted vs. achieved SE refraction in the TPRK group had a slope and coefficient (r^2^) of 0.996 and 0.9717, respectively, and 0.998 and 0.9594, respectively, in the off-flap Epi-LASIK group (shown in Fig. 1E).

**Fig. 2:**
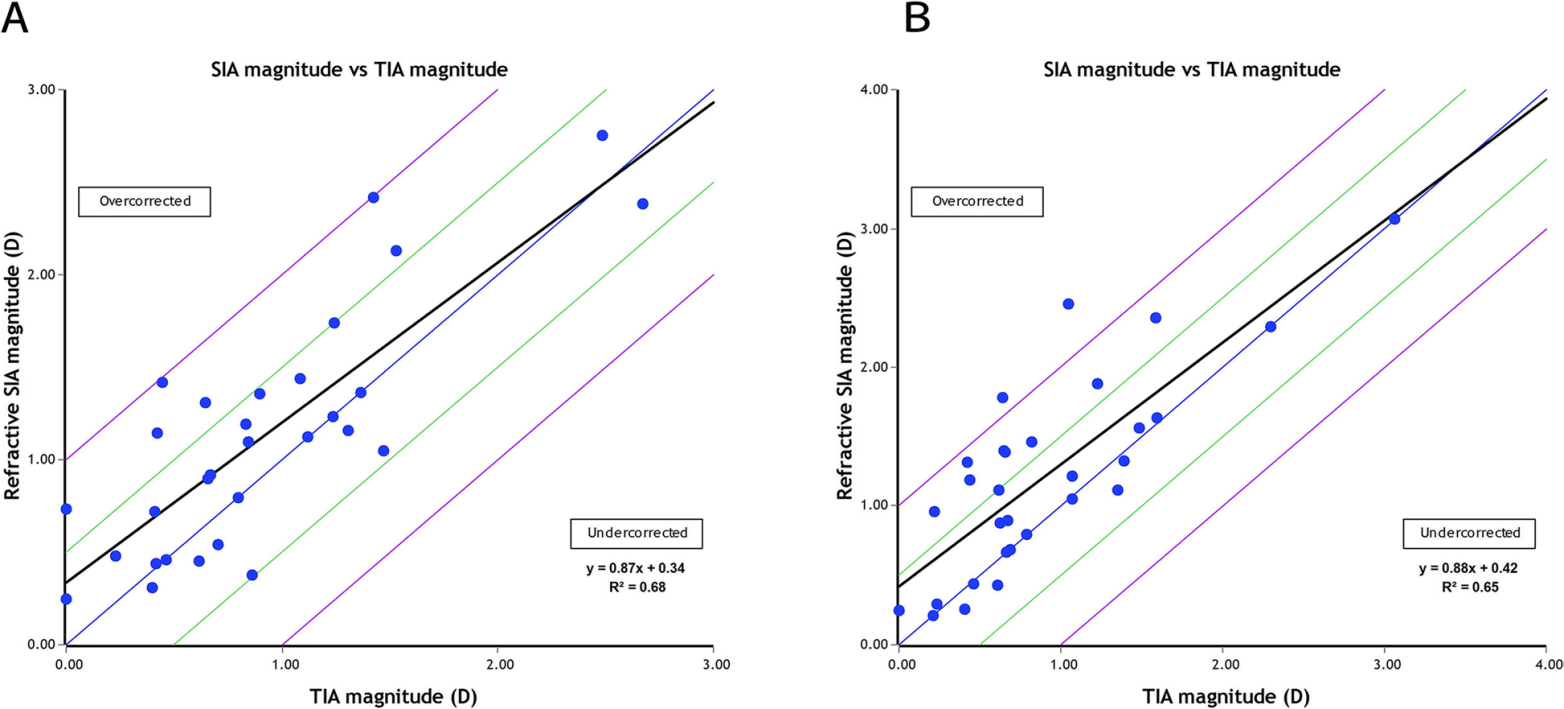
Target-induced versus surgically-induced astigmatism vectors at 12 months postoperatively

**Fig. 3:**
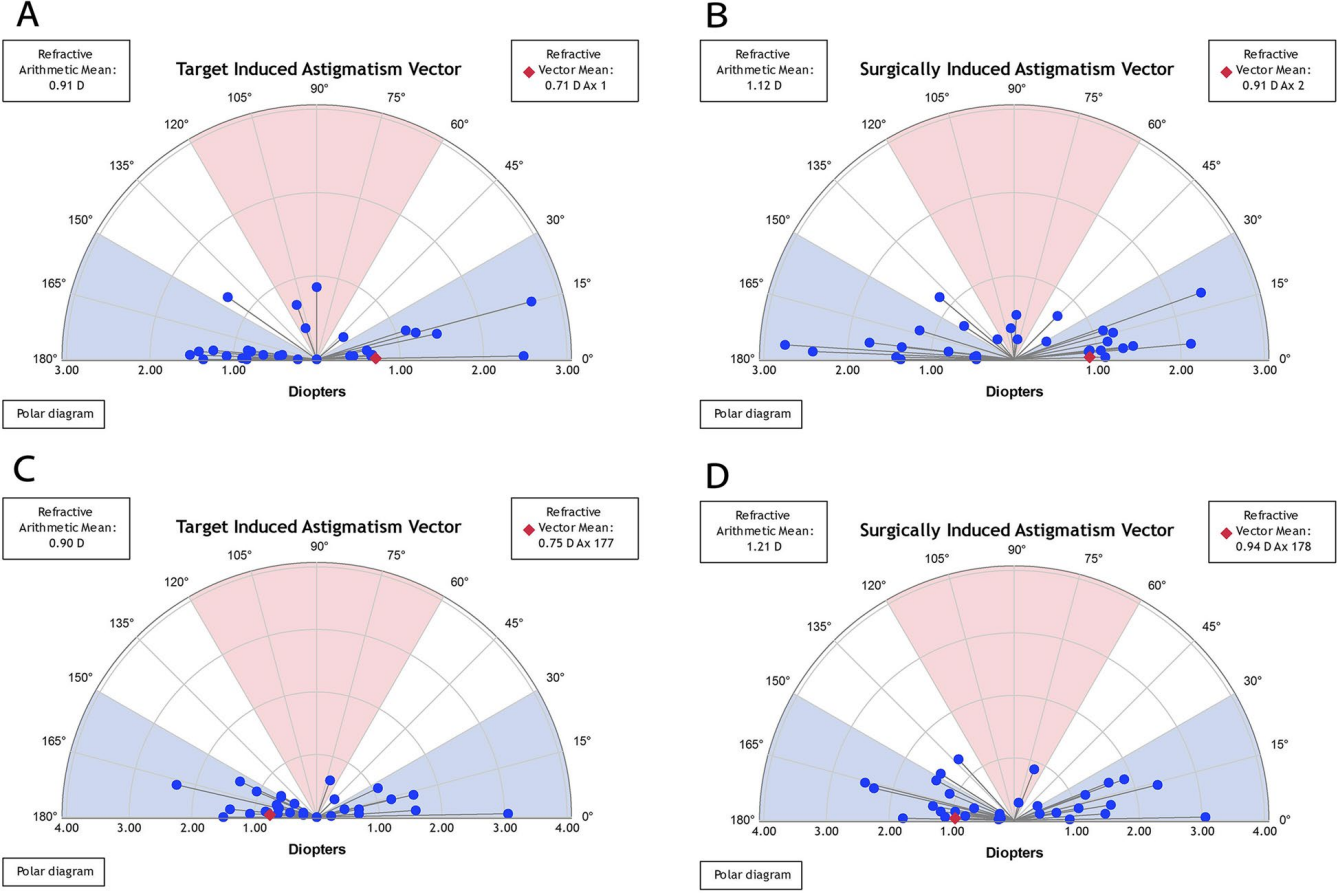
Single-angle polar plots of the target-induced astigmatism vector and surgically induced astigmatism at 12 months postoperatively in the single-step transepithelial photorefractive keratectomy (TPRK) group (**A**, **B**) and off-flap epipolis-laser in situ keratomileusis (Epi-LASIK) group (**C**, **D**), respectively

With respect to astigmatism correction, all treated eyes in the TPRK group and 31 treated eyes (97%) in the off-flap Epi-LASIK group exhibited postoperative cylinders of 1.00 D or less (shown in Fig. 1F). The linear regression analysis between the target-induced astigmatism (TIA) and surgically induced astigmatism (SIA) vectors revealed slopes and coefficients (r^2^) of, 0.87, and 0.68, respectively, in the TPRK group (shown in Fig. 2A) and 0.88, and 0.65, respectively, in the off-flap Epi-LASIK group (shown in Fig. 2B). Figure 3 shows the TIA and SIA vector polar diagrams of the TPRK group (shown in Fig. 3A and B) and the off-flap Epi-LASIK group (shown in Fig. 3C and D), respectively. No significant differences were found between two groups, with respect to magnitude and axis of TIA, SIA, difference vector and the value of angle of error, correction index and index of success (Table 3).

**Table 3:** Comparison of vector parameters between the TransPRK and off-flap Epi-LASIK groups

	Transepithelial PRK		Off-flap Epi-LASIK		*P* Value
Parameter	Mean ± SD	Range	Mean ± SD	Range	
TIA					
Magnitude (D)	0.91 ± 0.62	0.00, 2.67	0.90 ± 0.65	0.00, 3.06	.936
Axis (degrees)	102.0 ± 75.6	1.0, 180.0	109.2 ± 72.8	1.0, 180.0	.698
SIA					
Magnitude (D)	1.12 ± 0.65	0.25, 2.75	1.21 ± 0.71	0.21, 3.06	.464
Axis (degrees)	92.7 ± 75.5	1.1, 180.0	97.8 ± 74.7	1.0, 179.2	.796
Difference vector					
Magnitude (D)	0.43 ± 0.30	0.00, 1.00	0.48 ± 0.39	0.00, 1.49	.473
Axis (degrees)	81.4 ± 52.2	0.00, 170.0	82.2 ± 55.6	0.00, 175.0	.953
Angle of Error (degrees)	−1.5 ± 13.9	−38.9, 35.9	2.2 ± 13.2	−49.0, 72.1	.394
Correction index	1.32 ± 0.61	0.44, 3.19	1.45 ± 0.76	0.50, 3.90	.419
Index of success	0.53 ± 0.54	0.00, 1.99	0.65 ± 0.72	0.00, 3.05	.410

*TIA* Target induced astigmatism, *SIA* Surgically induced astigmatism

### Ocular wavefront aberrations

In the eyes of the TPRK group, there was a significant decrease in ocular spherical, coma, and trefoil aberrations (all *P* ≤ .027) 12 months after surgery. In the eyes of the off-flap Epi-LASIK group, there was a significant decrease in ocular spherical and trefoil aberrations (both *P* ≤ .012) 12 months postoperatively. The difference in ocular higher-order aberrations (HOAs) between the two groups was not significant at 12 months after surgery. By 12 months, the photopic and mesopic contrast sensitivity was not significantly different between the groups (Table 4).

**Table 4:** Comparison of ocular higher-order aberrations and contrast sensitivity in eyes that had transepithelial PRK or off-flap Epi-LASIK

	Transepithelial PRK		Off-flap Epi-LASIK		*P* Value
	Mean ± SD	Range	Mean ± SD	Range	
HOAs (um)					
Spherical	0.053 ± 0.120	0.001, 0.581	0.077 ± 0.206	0.001, 1.084	.348^*^
Coma	0.118 ± 0.200	0.007, 0.963	0.156 ± 0.245	0.007, 1.069	.209^*^
Trefoil	0.148 ± 0.205	0.019, 0.796	0.142 ± 0.150	0.021, 1.160	.802^*^
CS photopic					
3 cpd	1.76 ± 0.13	1.49, 1.93	1.74 ± 0.12	1.49, 2.08	.326
6 cpd	2.03 ± 0.16	1.70, 2.29	2.02 ± 0.17	1.70, 2.29	.670
12 cpd	1.73 ± 0.18	1.40, 1.99	1.72 ± 0.18	1.40, 1.99	.744
18 cpd	1.31 ± 0.18	0.96, 1.55	1.30 ± 0.18	0.81, 1.55	.689
CS mesopic					
3 cpd	1.73 ± 0.13	1.49, 1.93	1.70 ± 0.11	1.49, 1.93	.155
6 cpd	2.01 ± 0.14	1.70, 2.29	2.01 ± 0.17	1.70, 2.29	1.000
12 cpd	1.69 ± 0.19	1.40, 1.99	1.68 ± 0.20	1.40, 1.99	.714
18 cpd	1.25 ± 0.21	0.81, 1.55	1.26 ± 0.22	0.96, 1.55	.821

*HOA* Higher-order aberration, *CS* Contrast sensitivity. ^*^Mann-Whitney U test

### Haze

We discovered that one eye had 0.5 haze after off-flap Epi-LASIK and two eyes had 0.5 haze after TPRK. In the Off-flap Epi-LASIK group, 0.5 or 1.0 haze was noted in 19 eyes (59.4%), nine eyes (28.1%), and four eyes (12.5%) 1, 3, and 6 months postoperatively, respectively. In the TPRK group, 0.5 or 1.0 haze was found in 17 eyes (53.1%), 10 eyes (31.3%), and six eyes (18.8%) 1, 3, and 6 months postoperatively, respectively. There were no significant differences between the groups.

## Discussion

In this self-controlled study, we investigated the surgical outcomes of TPRK and off-flap Epi-LASIK in patients with moderate to high myopia. The visual outcomes indicated that both procedures were safe and effective for the correction of moderate to high myopia. There were no significant differences in postoperative visual acuity, efficacy, and safety indices as well as photopic and mesopic contrast sensitivity between the two groups. The ±0.50 D predictability for correction of the SE refraction was higher in the TPRK group than in the Off-flap Epi-LASIK group 12 months after surgery. Additionally, no significant differences in ocular HOAs (including spherical, coma, and trefoil aberrations) were noted between the groups.

Several previous studies have indicated that TPRK in myopic eyes is safe, effective and predictable [16, 17]. Xi et al. [18] reported that all of the eyes had postoperative UDVA 20/20 or better 6 months after TPRK. 63.8% of the eyes with residual refractive cylinder ≤ 0.25D, and 93.6% of the eyes ≤ o.50D. Another study found that refractive predictability to be higher in the TPRK procedure than the femtosecond LASIK procedure in high myopia [19]. Postoperative refraction was never more than ±1.00D in the TPRK eyes. Bartlomiej J et al. [20] evaluated refractive and visual outcomes after TPRK for the treatment mixed astigmatism with a large ablation zone, they observed 0.08D of hyperopic progression per year in 3-year follow-up, which limiting corneal regression. 79% eyes got postoperative UDVA within one Snellen line of preoperative CDVA 3 years after TPRK. Xi et al. [21]. reported that despite TPRK showed comparable visual results in low to high myopia, the postoperative UDVA was lower in the high myopia group than in low to moderate myopia groups 6 months after surgery. In eyes with high myopia, Ghadhfan F et al. [10] indicated that TPRK was significantly more likely to provide a final better UDVA than LASIK, LASEK and mechanical epithelial removal PRK. Agree with above studies, in our study, both TPRK and off-flap Epi-LASIK had comparable efficacy and predictability in the correction of moderate to high myopia. We included 32 patients (64 eyes) randomly had TPRK in one eye and off-flap Epi-LASIK in contralateral eye, none of the eyes lost two lines or more of CDVA in either group at the 12-month follow-up, and 60.00% (*n* = 18) of the eyes in the TPRK group and 63.33% (*n* = 19) eyes in the off-flap Epi-LASIK group gained one or two lines of improved CDVA. The ±0.50 D predictability for correction of the spherical equivalent (SE) was higher in the eyes of the TPRK group (91%) compared to those of the off-flap Epi-LASIK group (80%) 12 months after surgery. We showed better performance mainly because the included eyes almost with regular astigmatism less than 3.50 diopters, and amblyopia was excluded. Furthermore, TPRK has been modified several times since its introduction in 2009. Another study by Arba et al. [21] reported that if the actual corneal epithelial thickness is thinner or thicker than the applied profile and the actual corneal center-to-periphery progression deviates from the ablated one, refractive complications may occur in TPRK. In our study, TPRK demonstrated refractive results comparable to those of off-flap Epi-LASIK. While the reason for this is unclear, it may be that the eyes involved in our study were normal, non-pathologic, and not previously operated on; thus, these corneas highly matched the population-based epithelial ablation profile.

Regarding corneal haze, no significant difference was noted at any postoperative interval. By 6 months postoperatively, no eyes in either group had a haze over the score of 0.5. Haze can be seen after surface ablation in most cases as a mild anterior stromal opacity. It is considered to be the product of the normal healing process, which peaks at 3 months and fades at 6 months [22]. Haze formation is mainly associated with deep ablation, discontinuation of topical steroids [17], and irregularity of the anterior stroma [23]. The adjunction of 0.02% mitomycin C (MMC) over the ablated stroma led to lower levels of haze compared to conventional PRK, especially when correcting high myopia [24, 25]. The application of 0.02% MMC may explain why the eyes in these two groups did not experience severe haze. Furthermore, after removing the contact lens, tobramycin-dexamethasone drops were administered for the first 2 weeks, followed by 0.1% fluorometholone drops for the next 5 weeks to inhibit haze formation, and strictly follow up every month. All patients were required to wear sunglasses to prevent ultraviolet rays which may increase occurrence of haze.

In our study, TPRK eyes showed faster re-epithelialization compared to Epi-LASIK eyes, but no significant difference was noted which is similar to findings from previous studies. For example, Aslanides et al. [14] found that by day three, significantly more eyes that underwent TPRK (97%) had epithelial recovery compared to the alcohol-assisted PRK eyes (57%). Another study by Kaluzny et al. [26] used spectral OCT to evaluate the epithelium healing process in patients who underwent different procedures in their two eyes. They reported that the mean healing time was 2.4 ± 0.8 days in the TPRK group and 3.6 ± 0.9 days in the alcohol-assisted group. For the TPRK procedure, the epithelial ablation profile was based on the study by Reinstein et al. [27], which characterized the epithelial thickness of normal eyes. They found that this makes the ablation more precise, provides a smoother and more regular stromal surface as well as speeds up epithelium healing. Meanwhile, the diameter of the epithelial removal in TPRK matched the total ablation zone, which was significantly smaller than that in the off-flap Epi-LASIK (9 mm). This may explain why off-flap Epi-LASIK eyes needed a longer time to heal in our study.

In reflecting the quality of vision in daily life situations, more attention should be paid to contrast sensitivity and wavefront aberrations rather than visual acuity alone. Ocular high-order aberrations significantly decreased 12 months after surgery in both TPRK and off-flap Epi-LASIK groups, but the difference between the two groups was not significant. Corneal refractive surgery could correct low-order aberrations such as myopia and astigmatism, and improve UDVA, but several studies have reported the increasing amount of HOAs postoperatively and a high correlation between HOAs and visual performance [14, 28, 29]. Zheng et al. [30] reported significant induction of HOAs is general after refractive surgery. The creation of corneal flap, the stability of surgery laser energy, minor intraoperative eye movements, tear film quality, and corneal healing process may be the reasons for the increase of HOAs. Both TPRK and off-flap Epi-LASIK have no incision, thus the biomechanical properties of the cornea are better maintained. Besides, the stromal ablation was performed with Amaris 750 s excimer laser in two procedures, which improved residual stromal bed smoothness and reduced irregularity, and the usage of preservative-free artificial tears was required for at least 6 months to maintain tear film stability. These may explain why the HOAs decreased after TPRK and off-flap Epi-LASIK. Previous studies have demonstrated that surface ablation provides better visual quality than LASIK because it induces fewer HOAs than LASIK [31, 32]. In fact, higher-order aberrations are usually related to glare, halo, and vision disturbances, especially in patients with a large pupil or those under scotopic conditions. A certain correlation between the changes in corneal biomechanical properties after refractive surgery and the introduction of HOAs has been proved [33]. Wu et al. [34] reported that the value of index of surface variance (ISV) was lower after TPRK than that of femtosecond LASIK, which further indicated that TPRK had certain advantages over femtosecond LASIK in maintaining surface regularity and retaining the biomechanical properties of anterior surface. In terms of photopic and mesopic contrast sensitivities at 12 months postoperatively, we found no significant differences between TPRK and off-flap Epi-LASIK. For this result, we conclude that both TPRK and off-flap Epi-LASIK shared comperable performances not only in efficacy, safety, and predictability, but also in visual quality.

The current study had some limitations. First, the sample size was relatively small. A large population size could increase the power to detect significant differences. Second, the follow-up duration was relatively short. Therefore, future studies incorporating a longer follow-up of visual performance between TPRK and off-flap Epi-LASIK are recommended.

## Conclusions

We conclude that both TPRK and off-flap Epi-LASIK are safe, effective, and predictable for treating moderate to high myopia. These two types of surgical techniques have comparable epithelium healing time, pain scores, corneal haze grade, and visual outcomes.

## Supplementary Information

**Additional file 1.**

## Data Availability

All data generated or analysed during this study are included in this published article [and its supplementary information files].

## Abbreviations

TPRK: Single-step transepithelial photorefractive keratectomy
Epi-LASIK: Epipolis laser in situ keratomileusis
PRK: Photorefractive keratectomy
LASIK: Laser in situ keratomileusis
LASEK: Laser-assisted sub-epithelial keratectomy
CDVA: Corrected distance visual acuity
LogMAR: Logarithm of minimal angle of resolution
UDVA: Uncorrected distance visual acuities
CS: Contrast sensitivity
CCT: Central corneal thickness
HOAs: Higher-order aberrations
